# Supplementary material for: Morphological differentiation of peritumoral brain zone microglia
Source: PLoS One. 2024 Mar 7;19(3):e0297576. doi: 10.1371/journal.pone.0297576 (PMC10919594; doi:10.1371/journal.pone.0297576)
Supplement: S1 Fig — Number of Branches (NOB); Fractal dimension (FD); Lacunarity (LAC); Cell Area (CA); Convex Hull Area (CHA); Density (DEN); Cell perimeter (CP); Convex Hull Span Ratio (CHSR); Maximum span across the Convex Hull (MSACH); Convex Hull Perimeter (CHP); Roughness (R); Cell circularity (CC); Convex Hull Circularity (CHC); The ratio maximum/minimum Convex Hull radii (TRMM); Mean radius (MR); Diameter of the Bounding Circle (DOB). *P<0.05, **P<0.01, #P<0.001 (n = 80 cells/group). (DOCX) [file pone.0297576.s001.docx]

**
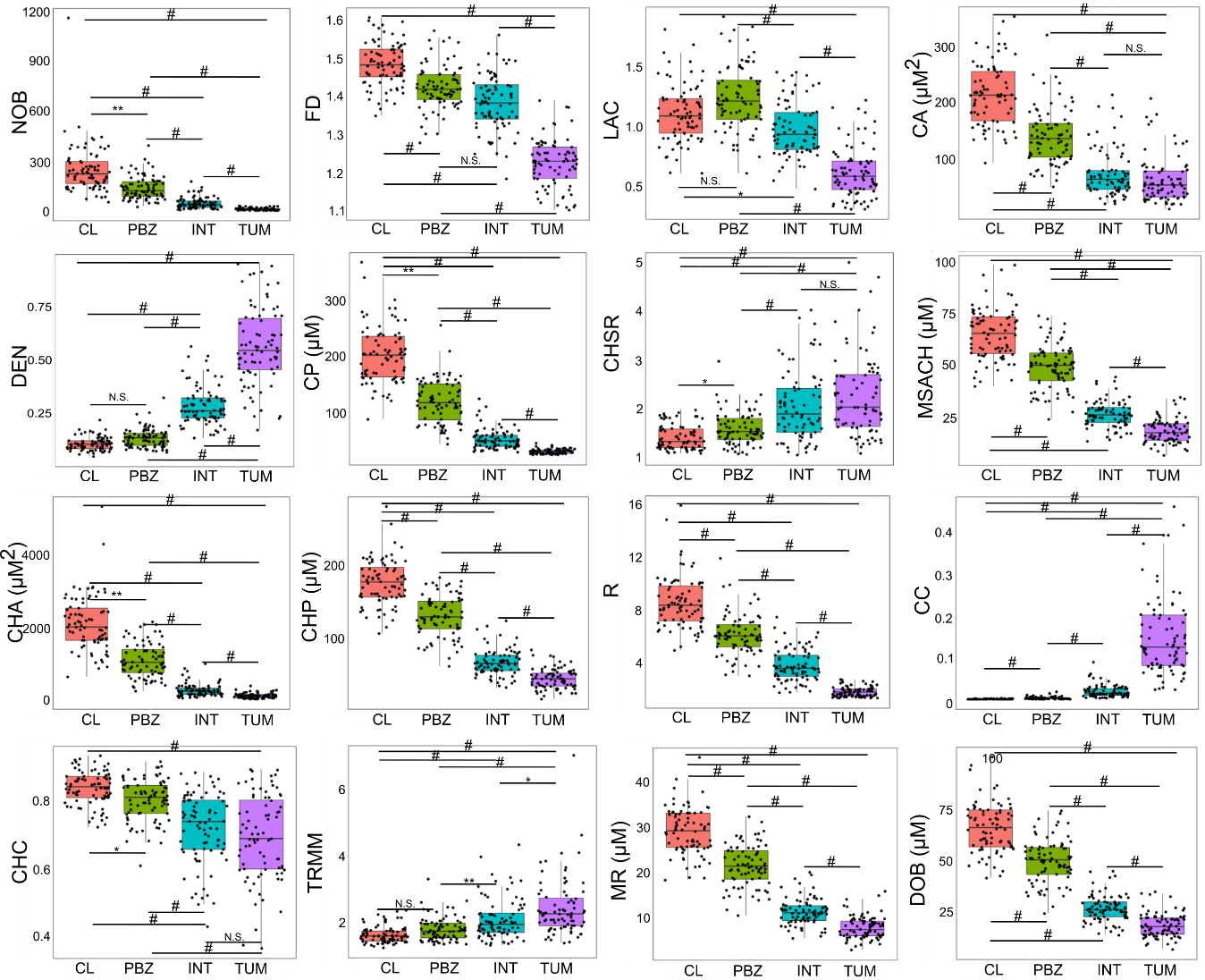
**

**Supplementary Figure 1. Comparison of the 16 morphological parameters of microglia sampled in the four regions of interest (T= tumor, I=interface, PBZ=peritumoral, CL=contralateral hemisphere)**. Number of Branches (NOB); Fractal dimension (FD); Lacunarity (LAC); Cell Area (CA); Convex Hull Area (CHA); Density (DEN); Cell perimeter (CP); Convex Hull Span Ratio (CHSR); Maximum span across the Convex Hull (MSACH); Convex Hull Perimeter (CHP); Roughness (R); Cell circularity (CC); Convex Hull Circularity (CHC); The ratio maximum/minimum Convex Hull radii (TRMM); Mean radius (MR); Diameter of the Bounding Circle (DOB). *P<0.05, **P<0.01, #P<0.001 (n=80 cells/group).
