# Supplementary material for: Morphological differentiation of peritumoral brain zone microglia
Source: PLoS One. 2024 Mar 7;19(3):e0297576. doi: 10.1371/journal.pone.0297576 (PMC10919594; doi:10.1371/journal.pone.0297576)
Supplement: S2 Fig — Significance matrix was built for the following parameters: Number of branches (NOB); Fractal dimension (FD); Lacunarity (LAC); Cell area (CA); Convex hull area (CHA); Density (DEN); Cell perimeter (CP); Convex hull span ratio (CHSR); Maximum span across the convex hull (MSACH); Convex hull Perimeter (CHP); Roughness (R); Cell circularity (CC); Convex hull circularity (CHC); Maximum/minimum convex hull radius ratio (TRMM); Mean radius (MR); Diameter of the bounding circle (DOB) comparing the four regions of interest (T = tumor, I = interface, PBZ, CL = contralateral hemisphere). P-values are provided and represented in a blue scale (n = 80 cells/group). (DOCX) [file pone.0297576.s002.docx]

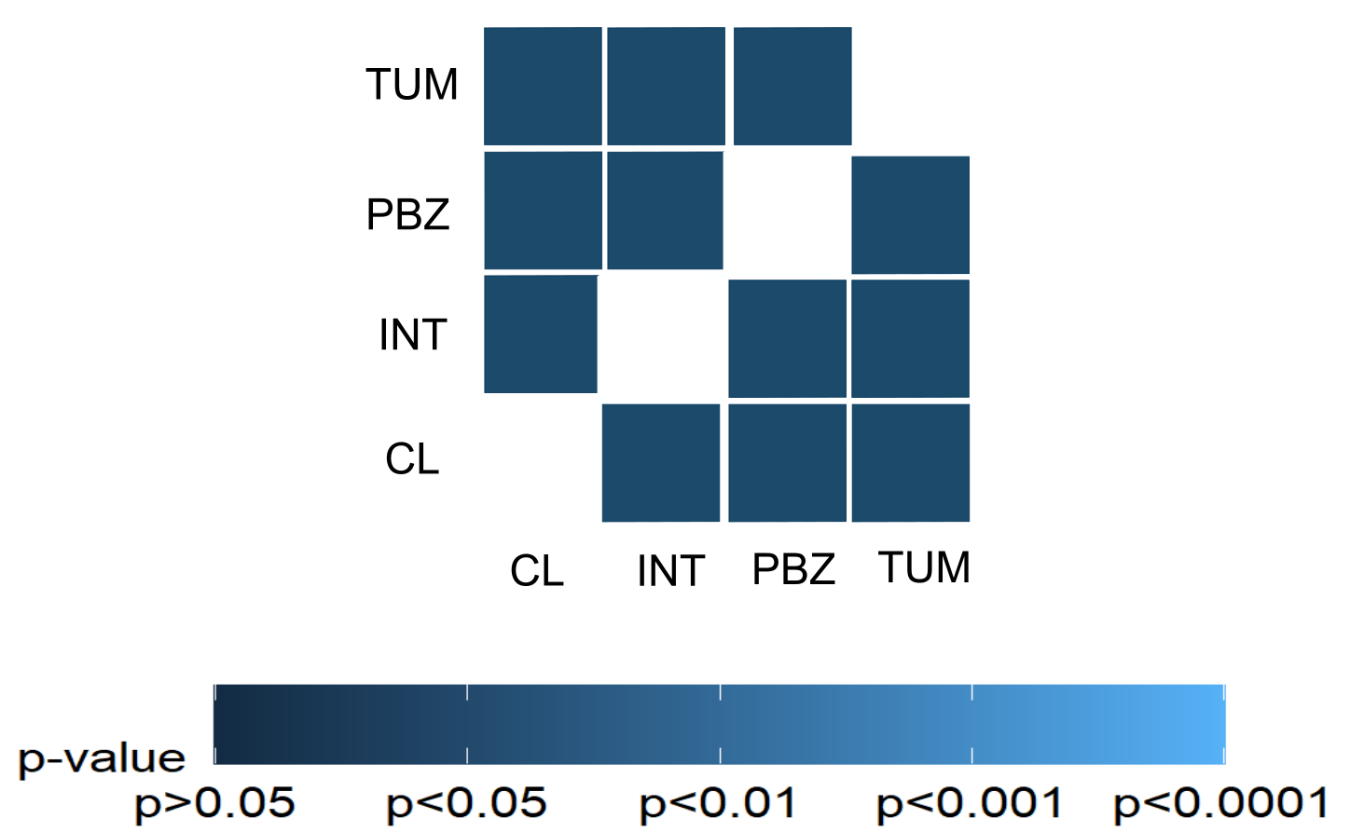


**Supplementary Figure 2. Multivariate comparisons among the sixteen morphological parameters measured in microglia sampled from the four ROIs.** Significance matrix was built for the following parameters: Number of branches (NOB); Fractal dimension (FD); Lacunarity (LAC); Cell area (CA); Convex hull area (CHA); Density (DEN); Cell perimeter (CP); Convex hull span ratio (CHSR); Maximum span across the convex hull (MSACH); Convex hull Perimeter (CHP); Roughness (R); Cell circularity (CC); Convex hull circularity (CHC); Maximum/minimum convex hull radius ratio (TRMM); Mean radius (MR); Diameter of the bounding circle (DOB) comparing the four regions of interest (T= tumor, I=interface, PBZ, CL=contralateral hemisphere). P-values are provided and represented in a blue scale (n=80 cells/group).
