# Supplementary material for: Morphological differentiation of peritumoral brain zone microglia
Source: PLoS One. 2024 Mar 7;19(3):e0297576. doi: 10.1371/journal.pone.0297576 (PMC10919594; doi:10.1371/journal.pone.0297576)
Supplement: S3 Fig — Number of Branches (NOB); Fractal dimension (FD); Lacunarity (LAC); Cell Area (CA); Convex Hull Area (CHA); Density (DEN); Cell perimeter (CP); Convex Hull Span Ratio (CHSR); Maximum span across the Convex Hull (MSACH); Convex Hull Perimeter (CHP); Roughness (R); Cell circularity (CC); Convex Hull Circularity (CHC); The ratio maximum/minimum Convex Hull radii (TRMM); Mean radius (MR); Diameter of the Bounding Circle (DOB). *P<0.05, #P<0.001. (DOCX) [file pone.0297576.s003.docx]

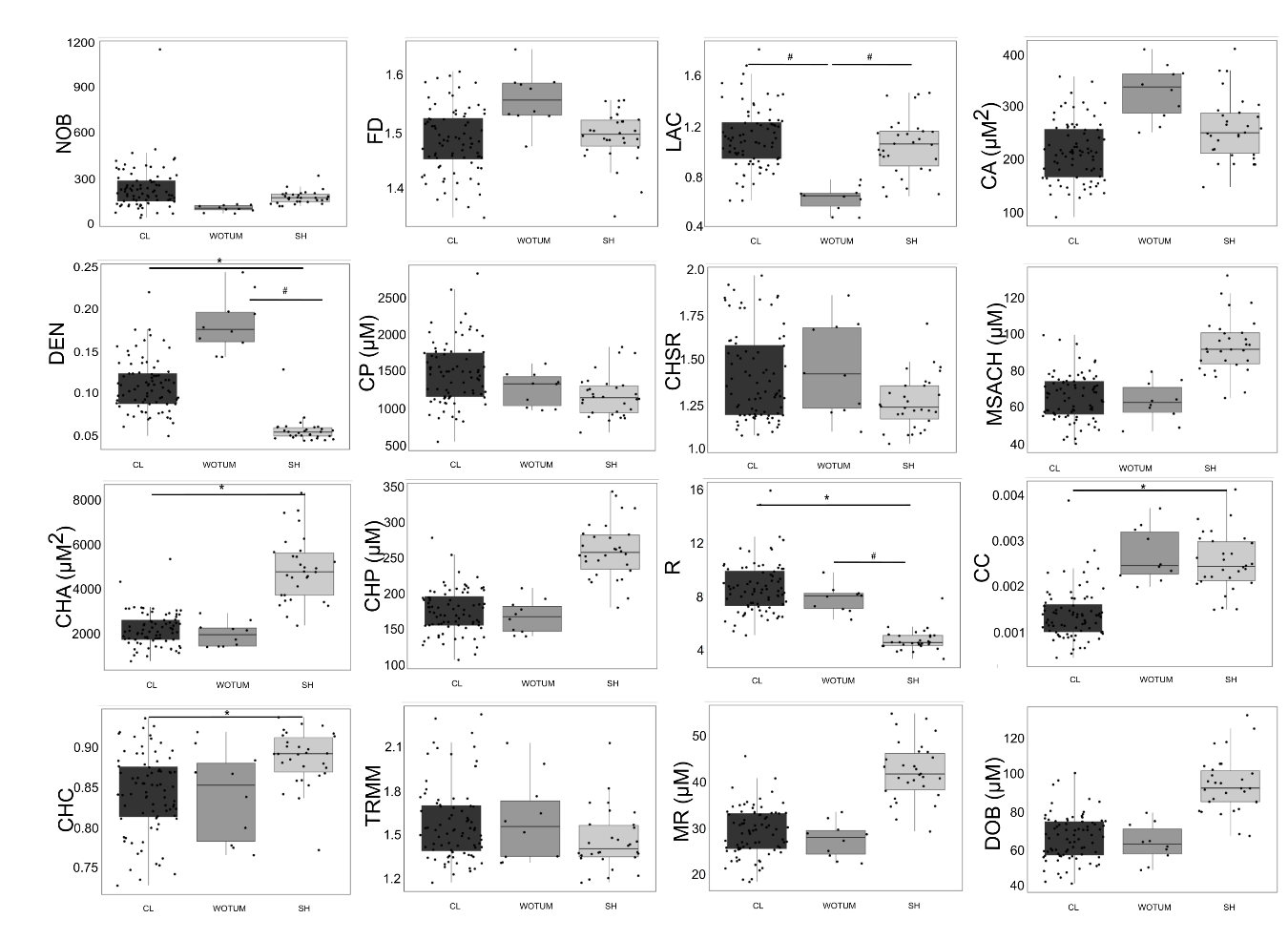


**Supplementary Figure 3. Comparison of the 16 morphological parameters of microglia sampled in two regions (CL=contralateral hemisphere, WOTUMOR=without tumor, SH=sham)**. Number of Branches (NOB); Fractal dimension (FD); Lacunarity (LAC); Cell Area (CA); Convex Hull Area (CHA); Density (DEN); Cell perimeter (CP); Convex Hull Span Ratio (CHSR); Maximum span across the Convex Hull (MSACH); Convex Hull Perimeter (CHP); Roughness (R); Cell circularity (CC); Convex Hull Circularity (CHC); The ratio maximum/minimum Convex Hull radii (TRMM); Mean radius (MR); Diameter of the Bounding Circle (DOB). *P<0.05, #P<0.001.
