# Supplementary material for: Morphological differentiation of peritumoral brain zone microglia
Source: PLoS One. 2024 Mar 7;19(3):e0297576. doi: 10.1371/journal.pone.0297576 (PMC10919594; doi:10.1371/journal.pone.0297576)
Supplement: S4 Fig — (A) Proportion of total variation contributed by the first principal components (PC). Note that only PC1, PC2 and PC3 contributed significantly to variance beyond the null distribution. (B) Contribution of each morphological parameters to PC1 definition for the actual values and for the null distribution. (C) Contribution of each morphological parameters to PC2 definition for the actual values and for the null distribution. (D) Contribution of each morphological parameters to PC3 definition for the actual values and for the null distribution. Number of Branches (NOB); Fractal dimension (FD); Lacunarity (LAC); Cell Area (CA); Convex Hull Area (CHA); Density (DEN); Cell perimeter (CP); Convex Hull Span Ratio (CHSR); Maximum span across the Convex Hull (MSACH); Convex Hull Perimeter (CHP); Roughness (R); Cell circularity (CC); Convex Hull Circularity (CHC); The ratio maximum/minimum Convex Hull radii (TRMM); Mean radius (MR); Diameter of the Bounding Circle (DOB). (DOCX) [file pone.0297576.s004.docx]

**
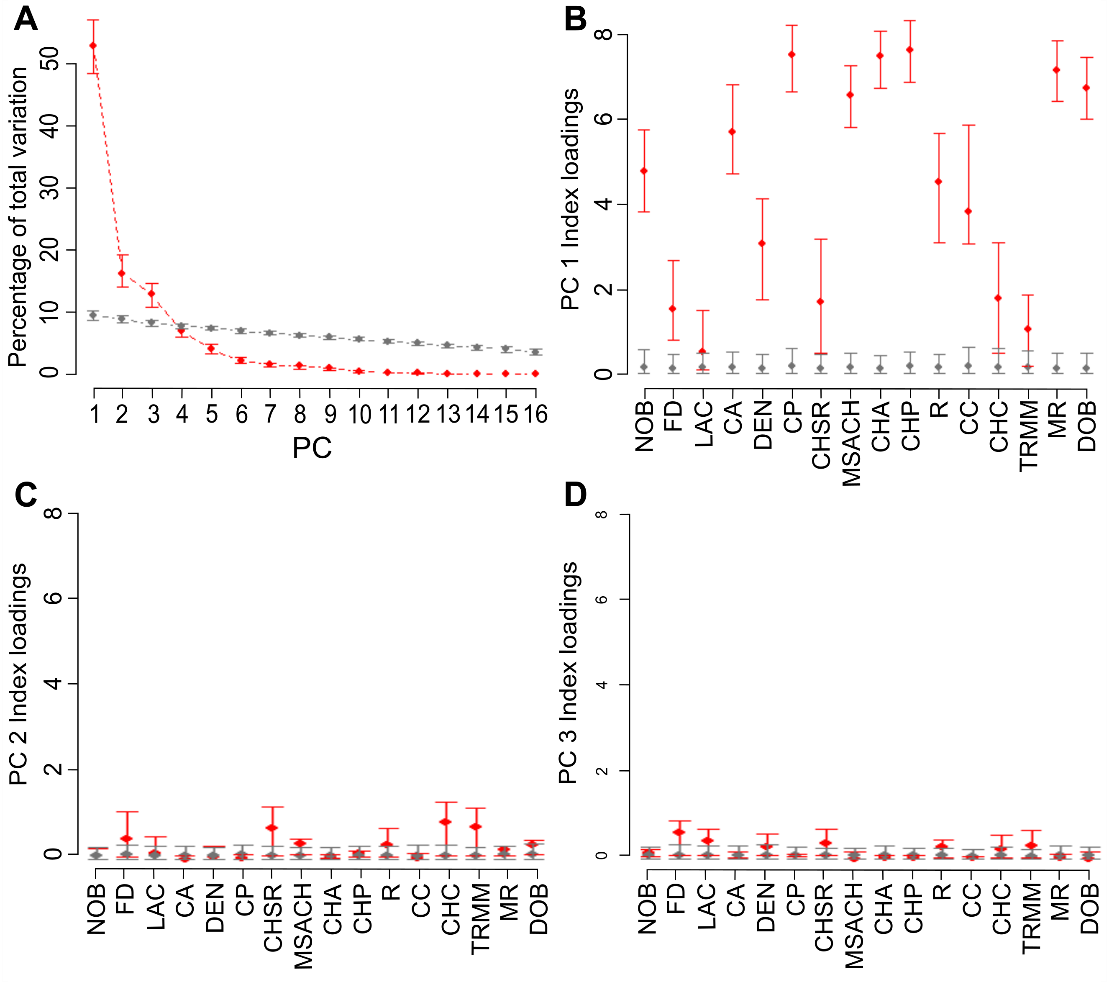
**

**Supplementary figure 4.** **Parameters defining Principal component analysis (PCA) of PBZ microglia.** **(A)** Proportion of total variation contributed by the first principal components (PC). Note that only PC1, PC2 and PC3 contributed significantly to variance beyond the null distribution. **(B)** Contribution of each morphological parameters to PC1 definition for the actual values and for the null distribution. **(C)** Contribution of each morphological parameters to PC2 definition for the actual values and for the null distribution. **(D)** Contribution of each morphological parameters to PC3 definition for the actual values and for the null distribution. Number of Branches (NOB); Fractal dimension (FD); Lacunarity (LAC); Cell Area (CA); Convex Hull Area (CHA); Density (DEN); Cell perimeter (CP); Convex Hull Span Ratio (CHSR); Maximum span across the Convex Hull (MSACH); Convex Hull Perimeter (CHP); Roughness (R); Cell circularity (CC); Convex Hull Circularity (CHC); The ratio maximum/minimum Convex Hull radii (TRMM); Mean radius (MR); Diameter of the Bounding Circle (DOB).
