# Supplementary material for: Morphological differentiation of peritumoral brain zone microglia
Source: PLoS One. 2024 Mar 7;19(3):e0297576. doi: 10.1371/journal.pone.0297576 (PMC10919594; doi:10.1371/journal.pone.0297576)
Supplement: Attachment — Submitted filename: Response to Reviewers.docx [file pone.0297576.s005.docx]

ANSWERS TO REVIEWERS, ORIGINAL EDITOR AND APPEAL (IN RED CAPITAL LETTERS):

Reviewer #1:

In this study authors provided objective and quantitative evidence that PBZ microglia represent a differentiable microglial morphotype that could contribute to the recurrence of GBM in this area. The idea of this study is interesting and adequate data has been provided. I highly recommend the acceptance of this article in its present form.

ANSWER: WE THANK THE REVIEWER (AS WELL AS THE OTHER REVIEWER) FOR THESE ENCOURAGING COMMENTS AND APPRECIATE HER EFFORTS REVIEWING OUR MANUSCRIPT.

I have only 2 comments as follows:
Since Figure 1B has also been shown in Figure 2A, it is recommended to merge Figure 1 into Figure 2.

ANSWER: FIGURES 1 AND 2 WERE MERGED, AS REQUESTED.

Please provide a lower magnification image for Ib1 IHC in Figure 2A.

ANWER: THE LOWER MAGNIFICATION IMAGE WAS INCLUDED, AS REQUESTED.

Reviewer #2:

In this paper, the authors perform a systematic morphological characterization of GBM peritumoral microglia in C6 cells implanted Sprague Dawley rats.
The aim is innovative and of considerable scientific interest. The authors point out that peritumoral microglia may have specific characteristics that differentiate them from both tumor-associated and healthy tissue microglia. This supports the importance of more specific studies directed not only at the tumoral but also at the peritumoral microenvironment as a possible player in the pathogenesis of tumor recurrence and other phenomena associated with tumor excitability (e.g., epileptogenesis).

ANSWER: WE THANK THE REVIEWER (AS WELL AS THE OTHER REVIEWER) FOR THESE ENCOURAGING COMMENTS AND APPRECIATE HER EFFORTS REVIEWING OUR MANUSCRIPT.

My only observation concerns line 886, where there is probably a typo (DEM instead of DEN).

ANSWER: THE TYPO WAS INDEED IN LINE 686 (INSTEAD OF LINE 886) AND WAS CORRECTED.

Original Editor:

I noticed that your study lacks an appropriate control (healthy) group.

ANSWER: SUCH INFORMATION WAS ALREADY INCORPORATED IN OUR ORIGINAL SUBMISSION. HOWEVER, WE ALSO INCLUDE THE DATA AS SUPPLEMENTARY FIGURE 2.

Additionally, no information was provided about the tumor development.

ANSWER: SUCH INFORMATION WAS ALREADY INCORPORATED IN OUR ORIGINAL SUBMISSION. HOWEVER, WE ALSO INCLUDE THE DATA IN NEW FIG. 1D.

Appeal:

i) We noted that tumor was allowed to develop for four weeks in the animals. Please specify the size of the tumour in mm3 at the end of the study.

ANSWER: SUCH INFORMATION WAS INCLUDE IN NEW FIG. 1D.

ii) Please clarify the method of sacrifice used in the study

ANSWER: SUCH INFORMATION WAS INCLUDE AT THE BEGINNING OF MATERIALS AND METHODS.

iii) We noted that the study was approved by the Bioethics Committee of the Institute of Neurobiology at UNAM. It is in our understanding that the bioethics committee typically handle human subject research. As the study involved animals, please could you clarify whether the study was also approved by an IACUC prior to experimentation.

ANSWER: THE BIOETHICS COMMITTEE OF THE INSTITUTE OF NEUROBIOLOGY AT UNAM EVALUATES OUR ANIMAL STUDIES AND FUNCTIONS AS AN IACUC. THIS INFORMATION WAS INCLUDE AT THE BEGINNING OF MATERIALS AND METHODS.

iv) Please provide a copy of the completed ARRIVE checklist.

ANSWER: INCLUDED AS ATTACHMENT.
