# Supplementary material for: Morphological differentiation of peritumoral brain zone microglia
Source: PLoS One. 2024 Mar 7;19(3):e0297576. doi: 10.1371/journal.pone.0297576 (PMC10919594; doi:10.1371/journal.pone.0297576)
Supplement: Attachment — Submitted filename: AnswerstoReviewers_FINAL.docx [file pone.0297576.s006.docx]

Responses

Editor:

Importantly, a control sham group is required to validate your findings.

Answer (A): We have incorporated the requested group and found that sham microglia are mostly undistinguishable from contralateral microglia and/or from microglia of tissue in which tumor was not developed (despite C6 cells were injected).

We note that you have included the phrase “data not shown” in your manuscript. Unfortunately, this does not meet our data sharing requirements.

A: We have deleted the expression “data not shown” and included the required information.

We note that Figures 1 and 2 in your submission contain copyrighted images.

A: Figures 1 and 2 DID NOT AND DO NOT contain copyrighted images.

Reviewer #1:

1. In Figure 1, we see that the rat gliomas are very discrete and not as infiltrative as typical human GBMs. This must be highlighted and discussed. Moreover, how these results may translate to human GBMs given this caveat needs further discussion.

A: Yes, the tumors were discrete and well-demarcated, likely due to the reduced number of injected cells and the host strain used (San-Galli et al., 1989), as has been found by others (Inoue et al. 1987, Farrell et al., 1987; San-Galli et al., 1989; Doblas et al., 2010; Giakoumettis et al., 2018). Indeed, magnetic resonance imaging show that C6 gliomas have a very clear sharpness in the transition from tumor to surrounding zones (Doblas et. al., 2010). While this actually was an advantage for our study, allowing us to consistently define the different zones for microglial characterization, we have acknowledged that the tumors obtained in this study do not exactly resemble advanced human GBMs (Inoue et al. 1987, Farrell et al., 1987; San-Galli et al., 1989; Giakoumettis et al., 2018). Perhaps our tumors only resemble early human GBMs (Inoue et al. 1987, Farrell et al., 1987; San-Galli et al., 1989; Giakoumettis et al., 2018). This information has been included in the Materials and Methods and the Discussion.

2. Further discussion of relevant literature is needed eg recent studies have corroborated reduced PBZ microglia compared to the GBM core, with differences in microglial markers between these regions including in pro-inflammatory and anti-inflammatory markers - these works need to be discussed in context of the findings presented here - PMID 37324244.

A: Relevant new literature has been incorporated. For instance: Pyonteck et al., 2013; Gieryng et al., 2017; Landry et al. 2020; Akkari et al., 2020; Noorani et al., 2023; Petterson et al., 2023 (look at the reference list ate the end of the answers).

3. Fig 1c is very low resolution - needs improved version.

A: We improved the resolution of the panel.

4. Fig 3 - the lettering / numbers are difficult to read - needs clarification. This is also true for Fig 4B, and supplementary figures 1 and 2.

A: We improved the lettering / numbers in all Figures mentioned.

5. It is unclear what subtype of GBM the authors are trying to model - it is known that different GBM molecular subtypes may contain different microglial patterns eg EGFR amplified vs non-amplified eg PMID: 30401716. Some discussion of this is warranted.

A: In this study we used the C6 cell line, which has been considered “the gold standard in glioma research” (Giakoumettis et al., 2018), because it reproduces GMB high growth rate and vascularization (Giakoumettis et al., 2018). C6 cells also express most markers found in human glioblastomas (Giakoumettis et al., 2018). Certainly, GBM and their cell lines are heterogeneous (Sibenaller et al., 2015; Gieryng et al., 2017; An et al., 2018; Giakoumettis et al., 2018; Mingzhi Zhang et al., 2019) and different GBM molecular identities differentially regulate microglial densities and phenotypes (An et al., 2018). For instance, GMB overexpressing the epidermal growth factor receptor variant III (EGFRvIII) favors microglial infiltration (An et al., 2018). This should be also the case for C6 cells since they also express EGFRvIII (Zhang et al., 2019), along with other genes such as PDGFβ, (IGF)-1, wild type EGFR, and Erb3/Her3 precursor proteins (for reviews see Barth and Kaur 2009; Giakoumettis et al., 2018). C6 also have a reduced expression of IGF-2, FGF-9, and FGF-10 while there is no change in the expression of MMP-7 gene (for reviews see Barth and Kaur 2009; Giakoumettis et al., 2018). A genomic comparison indicates that C6 cells exhibit high similarity with mesenchymal GBMs employing similar immune evasion strategies (Gieryng et al., 2017). This information has been included in the Discussion.

Reviewer #2:

-The number of rats used for each specific purpose, mortality rates, etc should be given in methods.

A: We have provided the requested information in the materials and methods.

Why authors did not create a sham surgery group in which nontumoral cell could be injected using identical procedures. It is important, since authors then could compare the effects of tumor with the effects of trauma induced by the needle. In fact, the main goal of authors is to describe microglia in the peritumoral zone, but with this design the changes reported can be caused by this mild trauma.

A: In our previous submission we included two controls: 1) the contralateral tissue as well as 2) animals without a tumor which directly deal with the issues mentioned by the reviewer. However, based on the reviewer’s suggestion, we now we have included sham tissue, as requested (now in Supplementary Figure 3).

“Then, it was transferred to a 30% sucrose solution until saturation.”
How authors define the saturation?

A: We defined saturation when the brain sank to the bottom of the 30% sucrose solution (Peña and Tapia, 1999; 2000; Tashiro et al., 2011). This information was included in the Materials and Methods section.

Non-specific binding is blocked by normal (bovine) serum, while tween and Triton X-100 are optional components of a solvent. Please, rephrase.

A: The sentence has been rephrased.

Please, specify manufacturer and reference for the secondary antibody.
A: The information has been included in Materials and Methods.

Please, describe the Nissl technique used.

A: We used cresyl violet staining (Peña and Tapia, 1999; 2000; Lorea-Hernández et al., 2016; Mendez-Salcido et al., 2022) as now indicated in Materials and Methods.

“Lemée et al. (2015) defined the PBZ as a radiologically normal peritumoral area, located within a 2 cm distance from the brain/tumor interface….”
“Then, MRI image was binarized and 2 mm from the tumor border were measured…”
Please, explain this transition from 2 cm to 2 mm.

A: We based this transition on the proportion between human brain mass (1508.91 ± 299.14; Azevedo et al., 2009) and rat brain mass (1.802 ± 0.313; Herculano-Houzel et al., 2006), which is 0.00119 and very close to the proportion used by us. This information has been incorporated in the Material and Methods section.

“To quantify the number of microglia in a 40x photomicrographs (25.96 x 103 μm2), seven slices (from four animals) were sampled to…”
This looks more like a density measurement. To quantify the number… one would need to use stereological counting and, in any case, 7 slices from 4 animals would not be sufficient.

A: Agree and have indicated that what we actually measured was microglial density (as already stated in the Y-axis of Fig. 1E).

“Eight cells per area (tumoral, inteface, peritumoral and contralateral), imaged at the highest magnification, were selected per slice.”

Please, give the criteria of the selection. These criteria should guarantee true randomness.

A: For this purpose, the 40x objective was randomly directed to each of the zones. All entire and nonoverlapping cells within the randomly selected area were selected, imaged at 100x and analyzed. When the area imaged with the 40x objective included more than the required eight cells, a random number generator was used to select the ones to be incorporated in the sample. This information has been incorporated in the Material and Methods section.

In statistics, why different tests (including normality tests) were used in each specific dataset should be explained. Many, if not all, morphological indices look intercorrelated. Therefore, MANOVA (or an equivalent test for nonparametric data) should be used to correct for multiple comparisons. For the very same reasons, the use of the regression analysis may be questionable. T-test may be too weak for post-hoc comparisons.

A: We aimed to apply the more adequate statistical tests based on the specific characteristics of any data set. However, we agree with the reviewer and performed the suggested analysis. We obtained identical results by performing a Non-Parametric MANOVA (NPMANOVA) using the *adonis* function from *vegan* package in RStudio Software (Noorani et al., 2023). All 16 measured parameters were evaluated for the 4 ROIs and significative differences were found (F(3)=262.99, p=0.001). A wrapper function for multilevel pairwise comparison using the *pairwise.adonis* function with Bonferroni correction was used as post-hoc test. The following results were obtained: CL vs PBZ (F(1)= 97.339504, p=0.01), CL vs INT (F(1)= 470.320998, p=0.01), CL vs TUM (F(1)= 723.741213, p=0.01), PBZ vs INT (F(1)= 194.567536, p=0.01), PBZ vs TUM (F(1)= 426.054392, p=0.01), INT vs TUM (F(1)= 81.390667, p=0.01). Regarding T-test, we only use it to compare the two types of microglia described in Fig, 7, which we think is appropriate in this case. The MPMANOVA comparisons were included in the results and as new Supplementary figure 2.

References

Akkari L, Bowman RL, Tessier J, Klemm F, Handgraaf SM, de Groot M, Quail DF, Tillard L, Gadiot J, Huse JT, Brandsma D, Westerga J, Watts C, Joyce JA. Dynamic changes in glioma macrophage populations after radiotherapy reveal CSF-1R inhibition as a strategy to overcome resistance. Sci Transl Med. 2020;12(552):eaaw7843.

An Z, Knobbe-Thomsen CB, Wan X, Fan QW, Reifenberger G, Weiss WA. EGFR Cooperates with EGFRvIII to Recruit Macrophages in Glioblastoma. Cancer Res. 2018 Dec 15;78(24):6785-6794.

Azevedo FA, Carvalho LR, Grinberg LT, Farfel JM, Ferretti RE, Leite RE, Jacob Filho W, Lent R, Herculano-Houzel S. Equal numbers of neuronal and nonneuronal cells make the human brain an isometrically scaled-up primate brain. J Comp Neurol. 2009 Apr 10;513(5):532-41.

Barth RF, Kaur B. Rat brain tumor models in experimental neuro-oncology: the C6, 9L, T9, RG2, F98, BT4C, RT-2 and CNS-1 gliomas. J Neurooncol. 2009 Sep;94(3):299-312.

Farrell CL, Stewart PA, Del Maestro RF. A new glioma model in rat: the C6 spheroid implantation technique permeability and vascular characterization. J Neurooncol. 1987;4(4):403-15.

Giakoumettis D, Kritis A, Foroglou N. C6 cell line: the gold standard in glioma research. Hippokratia. 2018 Jul-Sep;22(3):105-112.

Gieryng A, Pszczolkowska D, Bocian K, Dabrowski M, Rajan WD, Kloss M, Mieczkowski J, Kaminska B. Immune microenvironment of experimental rat C6 gliomas resembles human glioblastomas. Sci Rep. 2017 Dec 14;7(1):17556.

Herculano-Houzel S, Mota B, Lent R. Cellular scaling rules for rodent brains.Proc Natl Acad Sci U S A. 2006 Aug 8;103(32):12138-43.

Inoue T, Fukui M, Nishio S, Kitamura K, Nagara H. Hyperosmotic blood-brain barrier disruption in brains of rats with an intracerebrally transplanted RG-C6 tumor. J Neurosurg. 1987 Feb;66(2):256-63.

Landry AP, Balas M, Alli S, Spears J, Zador Z. Distinct regional ontogeny and activation of tumor associated macrophages in human glioblastoma. Sci Rep. 2020;10(1):19542.

Noorani I, Sidlauskas K, Pellow S, Savage R, Norman JL, Chatelet DS, Fabian M, Grundy P, Ching J, Nicoll JAR, Boche D. Clinical impact of anti-inflammatory microglia and macrophage phenotypes at glioblastoma margins. Brain Commun. 2023 2;5(3):fcad176.

Petterson SA, Sørensen MD, Burton M, Thomassen M, Kruse TA, Michaelsen SR, Kristensen BW. Differential expression of checkpoint markers in the normoxic and hypoxic microenvironment of glioblastomas. Brain Pathol. 2023 Jan;33(1):e13111.

Pyonteck SM, Akkari L, Schuhmacher AJ, Bowman RL, Sevenich L, Quail DF, Olson OC, Quick ML, Huse JT, Teijeiro V, Setty M, Leslie CS, Oei Y, Pedraza A, Zhang J, Brennan CW, Sutton JC, Holland EC, Daniel D, Joyce JA. CSF-1R inhibition alters macrophage polarization and blocks glioma progression. Nat Med. 2013 19(10):1264-72.

San-Galli F, Vrignaud P, Robert J, Coindre JM, Cohadon F. Assessment of the experimental model of transplanted C6 glioblastoma in Wistar rats. J Neurooncol. 1989 Sep;7(3):299-304.

Zhang M, Sun H, Deng Y, Su M, Wei S, Wang P, Yu L, Liu J, Guo J, Wang X, Han X, He Q, Shen L. COPI-Mediated Nuclear Translocation of EGFRvIII Promotes STAT3 Phosphorylation and PKM2 Nuclear Localization. Int J Biol Sci. 2019;15(1):114-126.
